# Individual and Group Response of Treatment with Ivacaftor on Airway and Gut Microbiota in People with CF and a S1251N Mutation

**DOI:** 10.3390/jpm11050350

**Published:** 2021-04-27

**Authors:** Maartje I. Kristensen, Karin M. de Winter-de Groot, Gitte Berkers, Mei Ling J. N. Chu, Kayleigh Arp, Sophie Ghijsen, Harry G. M. Heijerman, Hubertus G. M. Arets, Christof J. Majoor, Hettie M. Janssens, Renske van der Meer, Debby Bogaert, Cornelis K. van der Ent

**Affiliations:** 1 Department of Pediatric Pulmonology and Allergology, Wilhelmina Children’s Hospital—University Medical Center, Utrecht University, P.O. Box 85090, 3508 AB Utrecht, The Netherlands; m.i.kristensen-3@umcutrecht.nl (M.I.K.); k.m.dewinter@umcutrecht.nl (K.M.d.W.-d.G.); g.berkers-2@umcutrecht.nl (G.B.); s.c.ghijsen@students.uu.nl (S.G.); h.arets@umcutrecht.nl (H.G.M.A.); k.vanderent@umcutrecht.nl (C.K.v.d.E.); 2 Department of Pediatric Immunology and Infectious Diseases, Wilhelmina Children’s Hospital—University Medical Center, Utrecht University, P.O. Box 85090, 3508 AB Utrecht, The Netherlands; m.l.j.n.chu@umcutrecht.nl (M.L.J.N.C.); kayleigh.arp@rivm.nl (K.A.); 3 Department of Pulmonology, University Medical Center, Utrecht University, P.O. Box 85500, 3508 GA Utrecht, The Netherlands; h.g.m.heijerman@umcutrecht.nl; 4 Department of Respiratory Medicine, Amsterdam University Medical Center, P.O. Box 22660, 1100 DD Amsterdam, The Netherlands; c.j.majoor@amc.uva.nl; 5 Department of Pediatric Pulmonology, Erasmus Medical Center/Sophia Children’s Hospital, 3015 GD Rotterdam, The Netherlands; h.janssens@erasmusmc.nl; 6 Department of Pulmonology, Haga Teaching Hospital, 2545 AA The Hague, The Netherlands; r.vandermeer@hagaziekenhuis.nl; 7 The Queen’s Medical Research Institute, University of Edinburgh, Edinburgh EH16 4TJ, UK

**Keywords:** Cystic Fibrosis, microbiome, potentiator, airway, gut

## Abstract

Ivacaftor has been shown to restore the functionality of the S1251N (also known as c.3752G>A) mutated CFTR, which may cause alterations in both airway and gut physiology and micro-environment, resulting in a change of microbiota in these organs. The aim of the present study was to analyze the effects of ivacaftor on the microbial community composition of both airway and gut in subjects with CF carrying one S1251N mutation, using a 16S rRNA gene-based sequencing approach. In 16 subjects with CF, repetitive samples from airways and gut were collected just before, and 2 months after, and, for 8 patients, also 9 and 12 months after, start of ivacaftor. 16S rRNA based sequencing identified 344 operational taxonomical units (OTUs) in a total of 139 samples (35 nasopharyngeal, 39 oropharyngeal, 29 sputum, and 36 fecal samples). Ivacaftor significantly enhanced bacterial diversity and overall microbiota composition in the gut (*p* < 0.01). There were no significant changes in the overall microbial composition and alpha diversity in upper and lower airways of these patients after ivacaftor treatment. Treatment with ivacaftor induces changes in gut microbiota whereas airway microbiota do not change significantly over time.

## 1. Introduction

Mutations in CFTR can cause impaired chloride transport, dysregulated fluid balance, and thickened mucosal secretions in multiple organ systems [1,2], and are known to alter the airway and intestinal microenvironment. The human microbiome has increasingly been recognized as playing an important role in determining disease course and response to treatment. Previous studies indicated that both gut and respiratory microbiota of children with CF have lower bacterial diversity and are more unstable than those of healthy controls [3,4,5,6]. Several factors, such as infection, inflammation, nutrition, and antibiotic use, may cause changes or disruption of airway and gut microbiota [7,8,9].

It is likely that the microbiome of CF airways and gut will change as a result of CFTR modulation and the associated change in airway or gut physiology and micro-environment [10,11,12]. Ivacaftor has been shown to increase activity of defective cell-surface CFTR in vitro [10]. Treatment of CF patients with at least one gating mutation with ivacaftor led to significant improvement of sweat chloride secretion, lung function, and body mass index [13,14,15]. Most clinical studies of the effects of ivacaftor in subjects with CF have focused on conventional clinical and paraclinical outcome parameters [14,16,17,18]. Pathophysiological factors in CF which can influence airway microbiota composition include altered chloride transport resulting in dehydrated airways surfaces, decreased bicarbonate ion transport leading to dense immovable mucus, lowered pH in airway surface fluid disrupting innate immune function, and decreased oxygen concentration in obstructed airways [19]. We hypothesize that treatment with ivacaftor improves mucociliary clearance by inducing changes in ion fluxes and fluid balance. In this manner, it might also decrease infection and inflammation of these surfaces, which might lead to decreased use of antibiotics. Both of these mechanisms might cause a shift in the airway and gut microbiome.

The aim of the present study was to analyze the effect of treatment with ivacaftor on the microbial community composition of both the airway and gut in patients with CF carrying a S1251N (also known as c.3752G>A) gating mutation, using a 16S rRNA gene-based sequencing approach.

## 2. Materials and Methods

Sixteen patients with CF from four different CF centers (Rotterdam, Amsterdam, The Hague, and Utrecht) with at least one S1251N gating mutation who started treatment with ivacaftor, were monitored prospectively. They all received medical care according to standardized protocols. For each patient, nasopharyngeal (NP), oropharyngeal (OP), sputum, and fecal samples were collected one day before and two months after initiation of ivacaftor treatment, during routine visits in a CF outpatient clinic. In eight of these patients (all from Utrecht) follow up samples were also collected nine and twelve months after the start of treatment with ivacaftor.

This prospective multicenter observational study was approved by the medical ethics committee of our center or undertaken under approved protocol for the use of remaining material, in accordance with good clinical practice guidelines. All patients and/or parents in the case of patients who were minors gave written informed consent.

The posterior nasopharynx was swabbed transnasally [20] and the oropharynx transorally with an ESwab 482CE flexible or ESwab 493C02 regular-size nylon flocked sterile swab, respectively (Copan Diagnostics, Brescia, Italy), and stored in Amies transport medium [21]. All patients were experienced in expectorating sputum and all sputum samples were expectorated under the supervision of trained personnel (if necessary, with the help of a physiotherapist) to ensure that the expectoration technique was sufficient. Sputum samples were frozen within an hour. Fresh fecal samples were collected at home and stored at −20 °C, until they were taken to the outpatient clinic, where they were stored at −80 °C. Amies medium of both NP and OP samples was aliquoted. These aliquots, together with sputum and fecal samples, were stored at −80 °C until further sequence analysis was performed.

Bacterial DNA was isolated from all samples as previously described [22,23]. From respiratory samples 200 µL was used for isolation and from the fecal samples 20 µL was used. In each isolation run, control samples were included and only samples which had a 16S rRNA concentration of at least 0.3 pg/µL above the control samples from the same run were sequenced. Amplicon libraries of the V4 region of the 16S rRNA gene were constructed and sequenced using the Illumina Miseq platform (Illumina Inc., San Diego, CA, USA). Reads were trimmed and error corrections were performed using QIIME version 1.8 as previously described by Steenhuijsen Piters et al. [24]. All reads were assigned to Operational Taxonomical Units (OTUs) based on a 97% similarity. OTUs were taxonomically annotated based on the SILVA database. To check for contamination in the nasopharyngeal samples (which had low DNA concentrations), the Decontam package in R was used, correcting with both the frequency method and the prevalence method. Samples which contained less than 10.000 reads after correction for contamination were excluded from further analysis.

All statistical analyses were performed with R 3.3.0 in R-studio version 0.99.903. A non-metric Multidimensional Scaling (nMDS) plot (nMDS; vegan package) based on the Bray–Curtis dissimilarity was used to visualize dissimilarity between different niches. Differences between niches were analyzed using the permutation multivariate analysis of variance (PERMANOVA) test. Based on these results, further analysis was performed separately for all niches.

Alpha diversity (within sample bacterial diversity) was calculated for all samples with the Shannon index (Phyloseq package), which takes into account both bacterial richness and evenness. We hypothesized that the alpha diversity of follow-up samples would be higher after longer use of ivacaftor. Using a mixed effect model (lmerTest package), the effect of time on the alpha diversity was calculated for different niches.

Differences within niches over time were visualized with a nMDS plot based on the Bray–Curtis dissimilarity and analyzed with a PERMANOVA test. Additionally, unsupervised cluster-analysis of log-relative abundances based on the Bray–Curtis dissimilarity was performed to look at the effect of time after the start of ivacaftor treatment on relative microbial composition in different niches. In the literature, a decrease in relative abundance of CF-specific pathogens has been described [16,25,26]. A mixed effect model was used to study the effect of time after start of ivacaftor treatment at log-relative abundance of the top-15 keystone species per niche, corrected for the use of antibiotics. Residuals were not normally distributed for several OTUs. The Bonferroni method was used to correct for multiple testing.

## 3. Results

### 3.1. Baseline Characteristics

Baseline characteristics of the study population are summarized in Table 1.

For use of therapeutic and prophylactic antibiotics in the population studied during the study period, see Table S1. DNA was isolated from 156 samples, of which 139 samples (36 nasopharyngeal, 40 oropharyngeal, 29 sputum, and 36 fecal samples) contained sufficient DNA for downstream sequencing. 16S rRNA gene-based sequencing yielded a median of 37.176 reads per sample (range: 377–107.057), which were clustered in 344 OTUs. One nasopharyngeal and one oropharyngeal sample were excluded from further analysis due to insufficient read counts (<10.000 reads). Overall bacterial composition of the different niches is described in Figures S1 and S2.

**Figure 1:**
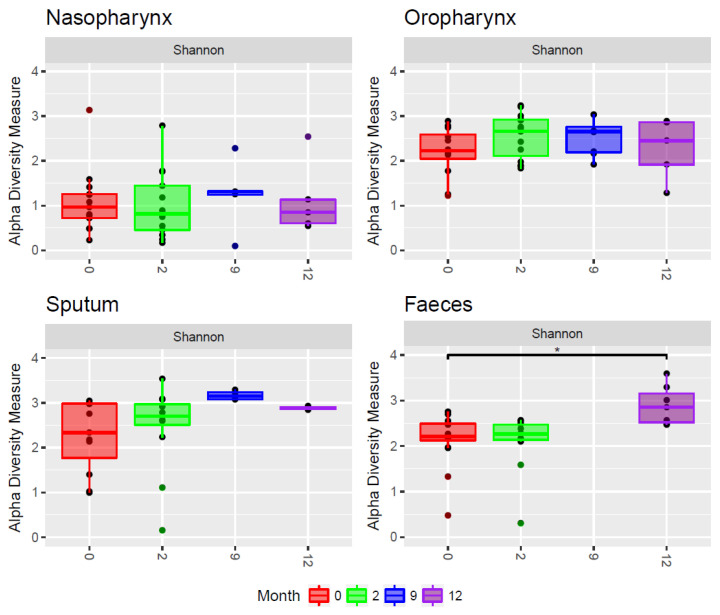
Alpha diversity calculated with the Shannon index for all different niches at T = 0 months (just before start of ivacaftor, red), and T = 2 months (green), 9 months (blue), and 12 months (purple) after start of ivacaftor. For fecal samples, the alpha diversity increases significantly with time after start of treatment (* *p* < 0.01). For sputum samples, the alpha diversity shows a trend towards increased diversity after start of ivacaftor.

With respect to diversity, nasopharyngeal samples showed the lowest alpha diversity compared to samples from other niches (Figure 1, Mann–Whitney test: *p* < 0.001).

**Figure 2:**
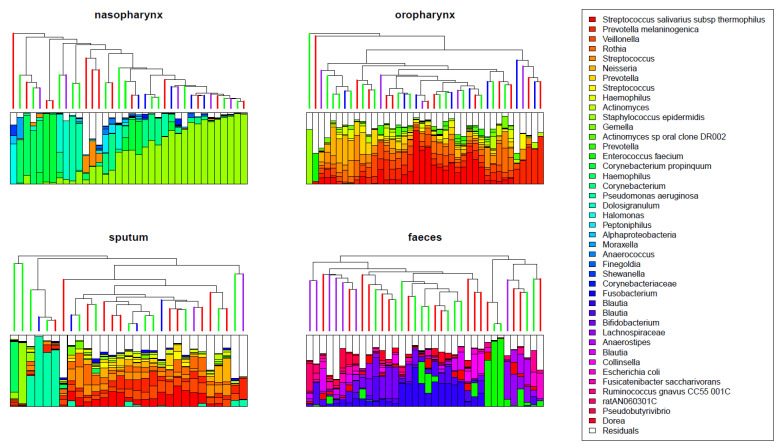
Dendrograms based on hierarchical clustering with the Bray–Curtis dissimilarity for all different niches. The colors of the branches of the dendrogram indicate the time point of the sample (red: T = 0 months, green: T = 2 months, blue: T = 9 months, and purple: T = 12 months). Both in oropharynx and in sputum samples, *Prevotella, Streptococcus, Veillonella*, and *Rothia* were the most abundant species. *Staphylococcus epidermidis, Corynebacterium, Haemophilus*, and *Pseudomonas* were most abundant in the nasopharynx, and fecal samples were dominated by *Bifidobacteria, Enterococcus*, and *Blautia*.

The most abundant bacteria in the nasopharynx were *Staphylococcus epidermidis*, followed by *Corynebacterium, Haemophilus*, and *Pseudomonas* spp. In oropharyngeal samples *Prevotella, Streptococcus, Veillonella*, and *Rothia* were most abundant; in sputum samples *Pseudomonas aeruginosa, Prevotella, Streptococcus, Veillonella*, and *Rothia*; and in fecal samples *Bifidobacteria, Enterococcus*, and *Blautia*. In sputum, we observed two distinct clusters: one cluster of samples dominated by bacteria also present in the nasopharynx, such as *Pseudomonas* and *Haemophilus*, and one cluster of samples with a microbial composition more similar to that of oropharyngeal samples (Figure 2).

### 3.2. Changes in Microbial Composition over Time during Treatment with Ivacaftor

#### 3.2.1. Gut

**Figure 3:**
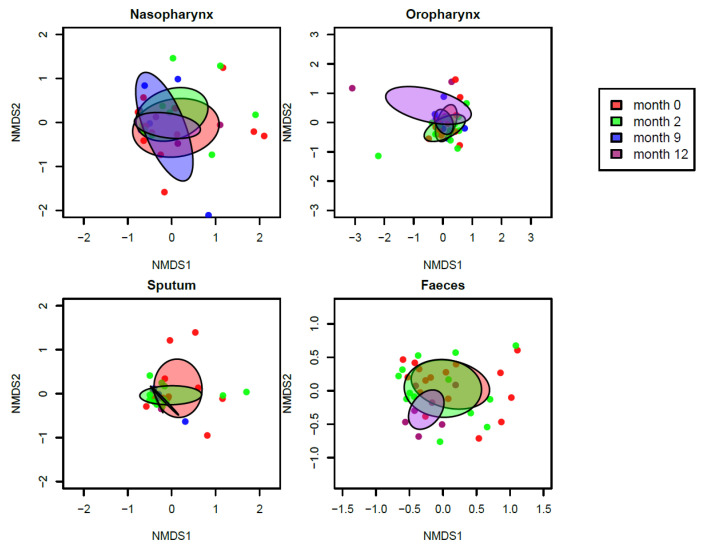
nMDS plots for nasopharyngeal, oropharyngeal, sputum, and fecal samples, colored per sampling moment, T = 0 months (red), 2 months (green), 9 months (blue), and 12 months (purple). Overall microbial composition in fecal samples was significantly different over time (*p* < 0.05).

In fecal samples we observed a significant increase in diversity over time after the start of ivacaftor treatment (linear mixed model: *p* < 0.01). In addition, the composition of fecal samples changed significantly over time (Figure 3, PERMANOVA test: *p* < 0.05). Samples obtained a year after start of treatment tended to cluster in the dendrogram and represented a more diverse community composition compared to time prior to treatment, (Figure 2). At the OTU level, no significant changes were found.

#### 3.2.2. Respiratory Tract

Although not significant, a trend towards increasing alpha diversity was also visible in sputum samples (*p* = 0.06). Interestingly, we found no significant changes in overall microbial composition of sputum samples over time (Figure 3). In the nasopharynx, we observed neither significant changes in alpha diversity nor in overall composition over time (Figure 2 and Figure 3). In the oropharynx, we hardly observed any effects of ivacaftor treatment, with no significant changes in alpha diversity in overall composition or at individual OTU level over time (Figure 2 and Figure 3).

## 4. Discussion

In this study we describe changes in the microbiome in four different niches after treatment with a CFTR modulator. Although the power of the study is limited, we can conclude that the most significant changes were observed in fecal samples. In contrast, changes in other niches, i.e., nasopharynx, oropharynx, and lung, were limited and the CF airway microbiome appeared to be very stable over time. However, baseline microbial composition may influence the effect of ivacaftor on the FEV1, although the power of this study was too limited to prove this.

Restoration of CFTR function due to ivacaftor might have an important positive influence on intestinal malabsorption, fluid balances, and nutritional status, and may lead to less need for antibiotic treatments. This might affect diversity and composition of the human microbiome in general. Exocrine pancreatic insufficiency in CF patients is mainly dependent on CFTR dysfunction [27]. Nielsen et al. [6] have already observed that patients with CF with better pancreatic function show higher gut microbial richness and diversity compared to patients with poor pancreatic function, suggesting a correlation between CFTR dysfunction, associated CF treatments, and gut microbiota diversity and function. These findings are supported by two small studies that found improvement of the exocrine pancreatic function in patients with a gating mutation after the start of ivacaftor [28,29]. Duytschaever et al. [3] reported a lower total richness for bacterial species in the gut in longitudinal samples of patients with CF compared to their healthy siblings. These studies support our finding that restoration of CFTR function by ivacaftor can increase microbial diversity.

Although a small increase in microbial diversity is observed in sputum, we do not find any changes in the overall microbial composition in sputum after the use of ivacaftor, nor in the other two upper respiratory tract niches.

Respiratory samples showed no significant changes in overall microbial composition in our study. However, because we noticed ivacaftor-related changes in the microbial community composition and structure of the gut, ivacaftor-induced effects, if at all present, appear to be less significant for these niches compared to the gut. The findings of Madan et al. suggest that development of respiratory tract microbiota is presaged by gut colonization patterns and nutritional factors [30]. It might therefore be that longer follow-up time is necessary to ultimately be able to detect significant changes in respiratory microbiota following ivacaftor treatment. Multiple other factors and interactions between microbiota might influence the respiratory microbiome composition, including CFTR genotype and environmental factors, such as presence of siblings, day care attendance, dietary intake, and age [27]. It is unknown how and in what direction these factors influence the microbial composition or diversity, and our study group was too small to correct for these factors.

Some previous studies have also assessed the impact of ivacaftor at the microbiota level. One recent four month placebo controlled crossover study found no differences between ivacaftor and placebo treatments in the change in airway microbiota composition in 20 adult patients [31]. Two earlier studies showed a small effect of ivacaftor treatment on the microbiome in sputum samples of CF patients carrying another gating mutation, namely G551D [16,26]. The first was performed by Bernarde et al. [26], who studied three children with CF carrying a G551D mutation. The authors also found that ivacaftor treatment for 3–5 months induces no major changes in CF sputum microbiota density and composition, but seems to enhance bacterial diversity. The second study, by Harris et al. [32], studied the microbiome in a group of 31 patients with a G551D mutation, and found no significant changes in microbial composition of the sputum after six months of ivacaftor treatment. They found a trend of increased abundance of *Prevotella*. However, CF patients in these studies carry a G551D mutation and their CFTR function may have responded differently to treatment with ivacaftor than patients carrying a S1251N mutation.

In our study we showed that, although all patients had the same mutation, baseline characteristics and baseline microbial composition differed greatly between patients, particularly in sputum samples. Differences in baseline sputum microbial profiles are likely due to differences in age, disease severity, use of antibiotics, and inflammation. In our small study, these different baseline microbial profiles were associated with differences in FEV1 at baseline. Although our study was too small to further investigate this, it would be interesting to look at differences of the effect of ivacaftor on clinical outcomes and microbial changes between patients with different baseline microbial profiles.

The small sample size is considered to be limitation of the present study. Furthermore, we note the overall small number of sampling time points and the short follow-up period. Unfortunately, samples after 6 months of treatment with ivacaftor were missing, but we collected the follow-up samples after 9 and 12 months. To evaluate the change in alpha diversity over time we used a linear mixed effect model with time as a continuous variable. Hence, we did not specifically test the difference in alpha diversity between M0 and M2. However, looking at Figure 3, the effect of increased alpha diversity in the gut seems to occur between month 2 and 12. We might expect this difference to occur earlier. However, there might be additional explanations for why this did not immediately become apparent, such as exacerbation, or (change in) antibiotic treatment. This limitation underlines the need for repeated sampling and analysis during follow up for future studies to better differentiate between short-term variation and long-term changes in microbial composition. Additionally, dietary changes during the first year of ivacaftor use could play an important role in the microbial changes in the gut. However, we did not investigate diet and dietary changes in this study. In the future, the effects of diet and dietary changes in relation to microbial changes after use of CFTR modulators should be taken into account. Future studies should also stratify patients according to genotype, because CF has a different penetrance for different mutations, which might affect changes in the microbiome. The study is too small to allow robust conclusions to be drawn; we therefore cannot state that ivacaftor has no effect on the airway microbiota of our CF patients, only that its effect, if at all present, is smaller than the effect of ivacaftor on fecal microbiota.

In conclusion, ivacaftor enhances bacterial diversity in the gut of subjects with CF with one S1251N mutation. We were unable to demonstrate significant and clinically relevant changes in the overall microbial composition and in alpha diversity in the upper and lower airways due to ivacaftor.

## Figures and Tables

**Table 1:** Characteristics of the study population. SD: standard deviation, BMI: body mass index, FEV1: forced expiratory volume in 1 s.

Number of Subjects	16
Cystic Fibrosis genotype (n, %)	
- S1251N/Phe508del	12 (75)
- S1251N/R117H	2 (13)
- S1251N/A455E	1 (6)
- S1251N/1717-1G > A	1 (6)
Age (years, mean±SD)	22.5 ± 12.8
Gender (n, %)	
- Female	5 (31)
- Male	11 (69)
Weight (kg, mean ± SD)	55.4 ± 21.7
Height (m, mean ± SD)	1.6 ± 0.2
BMI (kg/m^2^, mean ± SD)	19.9 ± 4.1
Pancreatic insufficiency (n, %)	13 (81)
FEV 1 (% predicted, mean ± SD)	76.4 ± 19.1
Microbiology, colonization	
- *Pseudomonas aeruginosa* (n, %)	8 (50)
- *Staphylococcus aureus* (n, %)	6 (38)

## Data Availability

The data presented in this study are openly available from the NCBI repository BioProject ID PRJNA698793.

## Supplementary Materials

The following are available online at https://www.mdpi.com/article/10.3390/jpm11050350/s1, Table S1: Use of antibiotics, Figure S1: Non-metric Multidimensional Scaling (nMDS) plot including all samples from all niches, Figure S2: Average microbial community composition per niche on phylum level.
