# Supplementary material for: Individual and Group Response of Treatment with Ivacaftor on Airway and Gut Microbiota in People with CF and a S1251N Mutation
Source: J Pers Med. 2021 Apr 27;11(5):350. doi: 10.3390/jpm11050350 (PMC8146888; doi:10.3390/jpm11050350)
Supplement: Supplementary file 1 [file jpm-11-00350-s001.zip › jpm-1170811-supplementary.docx]

**Table S1**. Antibiotic use during different time points of the study

| **Patient** | **Antibiotic therapy** |  |  |  | **Antibiotic prophylaxis** |  |  |  |
| --- | --- | --- | --- | --- | --- | --- | --- | --- |
|  | T=0 | T=0-2m | T=2-9m | T=9-12m | T=0 | T=0-2m | T=2-9m | T=9-12m |
| 1. | - | - | - | Co-trimoxazole | Co-trimoxazole | Co-trimoxazole | Co-trimoxazole | Co-trimoxazole |
| 2. | - | - | - | - | Azithromycin  Colistin neb | Azithromycin  Colistin neb | Azithromycin  Colistin neb | Azithromycin  Colistin neb |
| 3. | - | - | - | - | Tobramycin neb  Azithromycin | Tobramycin neb Azithromycin | Tobramycin neb Azithromycin | Tobramycin neb  Azithromycin |
| 4. | - | - | - | - | Tobramycin neb  Azithromycin | Tobramycin neb  Azithromycin | Tobramycin neb  Azithromycin | Tobramycin neb  Azithromycin |
| 5. | - | - | - | Ciprofloxacine | Azithromycin  Colistin neb | Azithromycin  Colistin neb | Azithromycin  Colistin neb | Azithromycin  Colistin neb |
| 6. | - | - | - | - | - | - | - | - |
| 7. | - | - | Azithromycin | - | Colistin neb  Co-trimoxazole | Colistin neb  Co-trimoxazole | Colistin neb  Co-trimoxazole | Colistin neb  Co-trimoxazole |
| 8. | - | - | Ciprofloxacine (2x) | Meropenem and Tobramycin | Tobramycin neb  Co-trimoxazole | Tobramycin neb  Co-trimoxazole | Tobramycin neb  Co-trimoxazole | Tobramycin neb  Co-trimoxazole |
| 9. | - | - |  |  | Colistin neb | Colistin neb |  |  |
| 10. | - | - |  |  | Colistin neb  Minocycline | Colistin neb  Minocycline |  |  |
| 11. | - | - |  |  | Co-trimoxazole | Co-trimoxazole |  |  |
| 12. | - | - |  |  | Colistin neb  Azithromycin | Colistin neb Azithromycin |  |  |
| 13. | - | - |  |  | Azithromycin  Tobramycin neb | Azithromycin  Tobramycin neb |  |  |
| 14. | - | - |  |  | - | - |  |  |
| 15. | - | - |  |  | - | - |  |  |
| 16. | - | - |  |  | Azithromycin  Tobramycin neb | Azithromycin  Tobramycin neb |  |  |

T=0: start of the study, m=months, neb: nebulization. Grey areas: not applicable

Supplemental materials figures: Overall presentation of different niches.


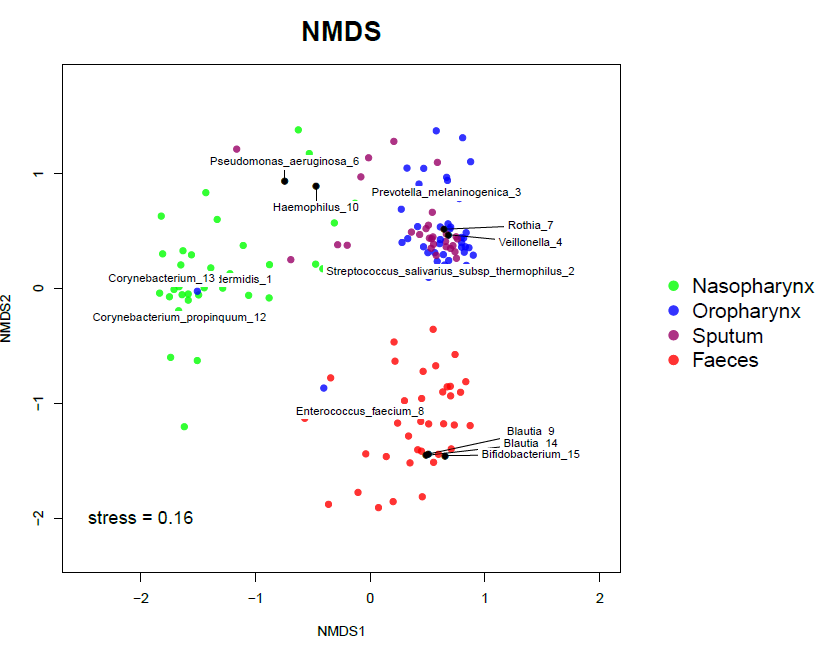
Nasopharyngeal, oropharyngeal, sputum and faecal samples significantly differ from each other in terms of overall microbial composition (PERMANOVA test: R^2^ 0.34, p < 0.001). The composition of oropharyngeal samples is most similar to that of sputum samples. Nasopharyngeal samples and faecal samples each have their distinct compositions, see Figure S1.

**Figure S1**. Non-metric Multidimensional Scaling (nMDS) plot including all samples from all niches. Microbial composition is significantly different per niche (p < 0.001). Oropharyngeal and sputum samples have the most similar composition. The four most abundant taxa per niche are plotted in the nMDS.


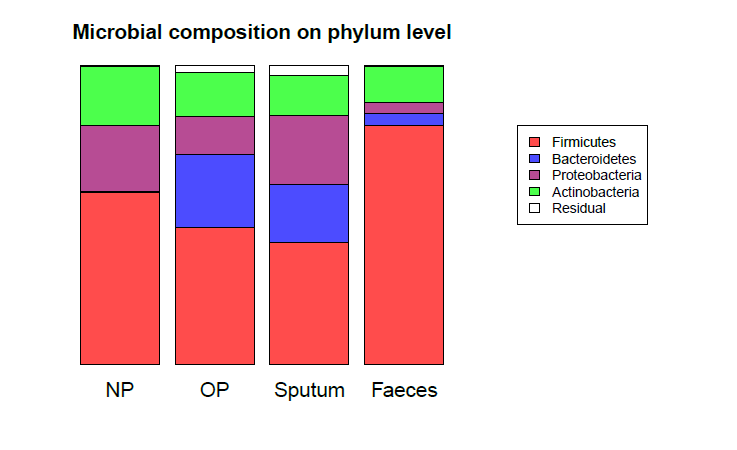
On phylum level, there are significant differences between niches in abundance of Firmicutes, Bacteroidetes and Proteobacteria (ANOVA: p < 0.001). Firmicutes is the most abundant phylum in all niches, but especially in faecal samples (80%). Bacteroidetes is seen most in oropharyngeal and sputum samples (25% and 19%, respectively), see Figure S2.

**Figure S2**. Average microbial community composition per niche on phylum level. Firmicutes is most abundant in all niches, but especially in faecal samples (80%). Bacteroidetes is second most abundant in oropharyngeal and sputum samples (25% and 19% respectively).
